# Expression, Location, Clinical Implication, and Bioinformatics Analysis of RNASET2 in Gastric Adenocarcinoma

**DOI:** 10.3389/fonc.2020.00836

**Published:** 2020-05-22

**Authors:** Zhi Zeng, Xu Zhang, Dan Li, Jin Li, Jingping Yuan, Lijuan Gu, Xiaoxing Xiong

**Affiliations:** ^1^Department of Pathology, Renmin Hospital of Wuhan University, Wuhan, China; ^2^Central Laboratory, Renmin Hospital of Wuhan University, Wuhan, China; ^3^Department of Pharmacy, Renmin Hospital of Wuhan University, Wuhan, China; ^4^Department of Biomedical informatics, The Ohio State University, Columbus, OH, United States

**Keywords:** RNASET2, early stage, gastric adenocarcinoma, bioinformatics analysis, biomarker

## Abstract

**Background:** In addition to exploiting its ribonuclease capacity, Ribonuclease T2 (RNASET2) has been reported to exert anti-angiogenic and anti-tumorigenic effects in several tumors. However, the role of RNASET2 in gastric adenocarcinoma (GAC) remains unclear. The purpose of this study was to explore the expression, location, and clinical implications of RNASET2 in GAC.

**Methods:** Data of RNASET2 mRNA expression in GAC and normal gastric mucosa tissues were extracted from three GSE series and 388 TCGA samples and reanalyzed. Genome-wide CRISPR/Cas9 proliferation screening datasets were used to investigate cell growth changes after RNASET2 knockout in 19 GAC cell lines. The biological processes involved in RNASET2 were studied by the bioinformatics analysis. Furthermore, the corresponding experiments including immunohistochemical staining, clinicopathological features analysis, survival curve, microvessel density detection, cell viability assay, and colony formation assay were performed to validate the expression and function of RNASET2 in GAC.

**Results:** An abundance of RNASET2 was present in the fundus glands and pylorus glands of the normal gastric mucosa. RNASET2 mRNA and protein were down-regulated in GAC compared with adjacent non-cancerous or normal gastric mucosa tissues. The expression of RNASET2 mRNA and protein in early GAC was higher than that in advanced GAC. 79/134 gene sets involved in the early GAC pathway were enriched in the RNASET2 mRNA high expression group. Genome-wide shRNA and CRISPR/Cas9 proliferation screening showed that knockdown or knockout of RNASET2 could not significantly promote GAC cell growth. AlamarBlue cell viability assay and colony formation assay in AGS cells further validated these results. Clinicopathologic features and survival analysis demonstrated that RNASET2 protein was significantly correlated with tumor cell differentiation, Lauren's classification, and TM4SF1 protein expression, but not correlated with lymph nodal metastasis and patient's prognosis. Microvessel density detection indicated that no significant correlation was found between the expression of RNASET2 protein and the angiogenesis of GAC.

**Conclusions:** Down-regulation of RNASET2 in GAC was only the consequence of the GAC, instead of the driver. The expression of RNASET2 could be regarded as a good biomarker for identifying the early stage of GAC.

## Introduction

Ribonucleases (RNases) are a large class of hydrolytic enzymes, involving a series of cellular processes such as DNA synthesis, RNA processing, and RNA degradation. These enzymes can be divided into endoribonucleases and exoribonucleases. The human ribonuclease T2 (RNASET2) gene, also known as RNASE6PL, belongs to the endoribonucleases class (1). It encodes a secreted glycoprotein (RNase T2) consisting of 256 amino acids and is a novel member of the Rh/T2/S-glycoprotein family (2, 3). RNASET2 is highly conserved among several species and plays various functions (4, 5). RNASET2, detected in the spermatozoa of male mouse and human, had the ability to inhibit the motility and progressive of sperm by coacting with actin network (6). It was involved in brain development and myelination through RNA metabolism or angiogenesis (7). Furthermore, overexpression of RNASET2 inhibited melanocyte outgrowth which might be related to the pathogenesis of vitiligo (8, 9).

In addition to its ribonuclease capacity, RNASET2 has been reported to exert anti-angiogenic and anti-tumorigenic effects in several tumor cell lines, such as colon cancer, breast cancer, and ovarian cancer (10, 11). Currently, RNASET2 was well-studied in ovarian cancer. RNASET2 protein expression was significantly decreased in a large proportion of ovarian tumor cell lines. Up-regulation of RNASET2 expression induced tumor cell senescence and inhibited the tumorigenicity of the ovarian tumor in nude mice model (1). Conversely, loss of function of RNASET2 promoted ovarian tumorigenesis (12). RNASET2 was also a tumor antagonizing gene in the melanoma model. It was reported that RNASET2 protein expression was reduced in several melanoma cells (13). Furthermore, genome-wide association analysis revealed a new locus of RNASET2 heterogeneity in genetic susceptibility associated with lung cancer risk, implying RNASET2 may function as a lung cancer suppressor gene (14). Besides the anti-tumorigenic activity, RNASET2 suppressed tumor cell motility and aggressiveness by binding cell actin and altering the cytoskeleton network structure (15). Up-regulation of RNASET2 protein significantly reduced the metastatic potential of ovarian cancer (11). Finally, down-regulation of RNASET2 protein was associated with drug resistance in ovarian cancer (16).

In physiological contexts, the top five RNASET2 mRNA-rich organs in human body are spleen, colon, lymph node, bone marrow, and stomach (17), which indicates that RNASET2 may be involved in the key biological processes of lymphohematopoietic organs and the gastrointestinal tract. Several recent reports confirmed that RNASET2 plays an important role in regulating the immune response by manipulating macrophage recruitment and polarization (18). In addition, the role of RNASET2 in colon cancer has also been reported. Compared to control cells, mouse RNASET2-expressing C51 colon cancer cells showed strong delayed tumor growth (19). And human recombinant RNASET2 disrupted angiogenesis and inhibited clonogenicity of colon cancer cells (20, 21). Until now, the physiological and pathological roles of RNASET2 in the stomach remain unclear. Accordingly, we investigated the expression and function of RNASET2 protein and mRNA in the normal gastric mucosa and gastric adenocarcinoma (GAC) tissues, as well as GAC cell lines, by immunohistochemical staining, microvessel density detection, cell viability assay, colony formation assay, and bioinformatics analysis. The relationships between RNASET2 expression and clinicopathological features, patient's prognosis, GAC-associated markers, as well as various biological processes, were further analyzed.

## Materials and Methods

### Tissue Sample and Data Collection

Eighty-two cases of endoscopic biopsy normal gastric mucosa tissues (54 males and 28 females; age range, 36–65 years old; average age 52.45 ± 8.00 years old) were collected from healthy volunteers who visited the health check-up center for their routine scheduled physical exams. One-hundred and sixty-eight cases of GAC tissues (109 males and 59 females; age range, 23–84 years old; average age 56.04 ± 10.23 years old; tumor size range, 0.5–10 cm; average size 4.14 ± 2.39 cm, 15 early GAC and 153 advanced GAC) were included in the second cohort. To investigate the expression of RNASET2 protein in the primary tumor, metastases, and paired adjacent non-cancerous gastric mucosa tissues, another 27 cases of advanced GAC patients with lymph nodal metastasis (17 males and 10 females; age range, 31–78 years old; average age 60.48 ± 10.05 years old; tumor size range, 0.5–8.0 cm; average size 3.99 ± 2.23 cm) were enrolled in the third cohort. Samples in the second and third cohorts were surgical resection tissues for GAC. In order to increase the number of early GAC, 20 cases of TNM IA stage GAC tissues (13 males and 7 females; age range, 50–69 years old; average age 60.05 ± 5.54 years old) were randomly selected from endoscopic submucosal dissection (ESD) specimens. Therefore, A total of 35 cases of early GAC were involved in this study. All tumor cases were identified by gastrointestinal surgery and pathology department, and through cancer registries in the Renmin Hospital of Wuhan University between January 2009 and December 2015. Histopathological diagnosis confirmed that 100% of the cases were GAC. After giving written consent, clinicopathological features including age, sex, size of the tumor, depth of invasion, nodal metastasis, cell differentiation, Lauren's classification, and TNM stage were collected from medical records of the hospital. In the present study, TNM stage was confirmed according to the Union for International Cancer Control (UICC) classification (8th edition) (22).

For survival analysis, 117 cases of GAC patients from the second cohort, who received the same surgical resection without preoperative radiotherapy or chemotherapy, were followed up. The follow-up time was ended on April 17, 2017, and the information of survival status was obtained from clinic records and patient or family contacts. The duration of overall survival was recorded as the time interval between the date of surgical resection and the last known date alive or dead. This study was approved by the ethics committees of Renmin Hospital of Wuhan University.

### Immunohistochemistry (IHC)

Formalin-fixed and paraffin-embedded sections (4 μm) were obtained for immunohistochemical staining according to the standard protocol. Briefly, deparaffinized and rehydrated sections were treated with 3% H_2_O_2_ and subjected to antigen retrieval by citrate buffer (pH 6.0). Then, sections were blocked with 5% BSA for 20 minutes and incubated overnight (16–18 h) with primary antibody (Anti-Ribonuclease T2 antibody, 1:100, ab107313, anti-TM4SF1, 1:200, ab113504, and anti-S100A12, 1:250, ab37657, Abcam, Cambridge, MA, USA; anti-NF-κB p65, 1:100, sc-109, Santa Cruz Biotechnology, Santa Cruz, CA, USA; anti-ERK, 1:250, #4695, Cell Signaling Technology, Beverly, MA, USA; Anti-CD34 [MAB-0034, QBEnd/10], anti-Ki67 [MAB-0129, MIB-1], and anti-p53 [MAB-0142, DO-7], Ready-to-use; Maixin Bio, Fujian, China) at 4°C. Then they were incubated with biotinylated linked antibodies and peroxidase-labeled streptavidin (UltraSensitive^TM^ SP (Mouse/Rabbit) IHC Kit-9710; Maixin Bio, Fujian, China) for 15 min each at room temperature. The reaction products were stained with 3,3′Diaminobenzidine (DAB) and slight counterstained with hematoxylin. The sections with PBS, replacing the primary antibody, and served as negative control.

### Evaluation of Immunohistochemical Staining

The immunohistochemical staining results of RNASET2, TM4SF1, S100A12, ERK, NF-κB p65, p53, Ki67, and CD34 in all sections were evaluated by two independent pathologists (ZZ and JY) who were unaware of the diagnosis outcome. In the present study, the RNASET2 protein was mainly located in the tumor cytoplasm and presented in a different staining intensity, hence, the expression level of RNASET2 protein was classified according to a four-tier grading system (scores: 0 = absent, 1 = weak, 2 = moderate, and 3 = strong staining intensity). In addition, cases with the percentage of RNASET2 staining-positive cells <5% were also considered as score 0. To analyze, RNASET2 protein expression level was further divided into two groups: Negative Group (score value = 0 or 1) and Positive Group (score value = 2 or 3). Proteins of NF-κB p65 and ERK were observed in the cell nucleus and cytoplasm. However, ERK and NF-κB p65, located in the nucleus, represented the form of its activation. Hence, the percentage of nucleus staining-positive cells of the two proteins in the tumor tissues were analyzed as the IHC score and were divided into two groups: Negative Group <10%; Positive Group ≥ 10%. TM4SF1, S100A12, p53, and Ki67 protein expression levels were evaluated as our previous studies (23, 24). Relative microvessel density, defined as the mean number of blood vessels presented in the same measured area of tissue, was calculated using the “hot spots method.” In this study, we used the antibody of CD34 to label the blood vessels and selected three areas with the highest number of blood vessels under low magnification (100×). Then the number of microvessels were counted under a magnification of 400× in a selected area. The arithmetic means of the three “hot spots” was calculated for relative microvessel numbers.

### Cell Culture and SiRNA Transfection

Human GAC cell line (AGS) was purchased from the Cell Bank of the Shanghai Institute for Biological Science (Shanghai, China) and cultured in F-12K (Hyclone, Thermo Fisher, USA) medium supplemented with 10% fetal bovine serum (FBS; Hyclone, Thermo Fisher, USA) and 1% penicillin/streptomycin (Invitrogen, Carlsbad, CA, USA) at 37°C in a humidified atmosphere with 5% CO_2_. All cells used in the experiments were in the exponential growth phase. RNASET2 siRNA and negative control (NC) siRNA were chemically synthesized (Thermo Fisher Scientific, Massachusetts, USA). For each transfection, 30 pmol per well (6-well-plates) of RNASET2 siRNA or NC siRNA was, respectively, transfected into AGS cells using Lipofectamine® RNAiMAX Reagent (Invitrogen, Carlsbad, CA, USA) according to the manufacturer's instructions.

### Nucleic Acid Preparation and Reverse Transcription-polymerase Chain Reaction (RT-PCR)

Total RNA was harvested from AGS cells using PureLink^TM^ RNA Mini kit (Life Technologies, Austin, TX, USA), and cDNA was synthesized from 1 μg total RNA using iScript^TM^ cDNA Synthesis kit (BIO-RAD, Hercules, CA, USA) according to the manufacturer's instructions. RNASET2 gene were amplified using AmpliTaq Gold® 360 Master Mix (Thermo Fisher Scientific, Massachusetts, USA). The primer sequences were: RNASET2 forward (5′-AATCCAGTGCCTTCCACCAAGC-3′) and RNASET2 reverse (5′-CCATTTGCCAGCCAGACTTCCT-3′). The thermal cycling profile for PCR was set up as follows: pre-denaturation at 95°C for 10 min, 32 cycles of denaturation for 30 s at 95°C, annealing for 30 s at 58°C, and extension for 15 s at 72°C, followed by a final extension at 72°C for 7 min. PCR products size of RNASET2 was 150 bp. 18S ribosomal RNA (RN18S1) gene was served as internal control for normalization of RT-PCR data.

### *In vitro* Cell Proliferation Assay

For the cell viability assay, the AGS cells were trypsinized after siRNA transfection of RNASET2 for 48 h, and then 2.5 × 10^3^ cells per well were plated into 96-well-plates with each well containing 100 μl medium. After cultured for additional 0.5, 2, and 3 days, the AGS cells were stained using an AlamarBlue HS Cell Viability Reagent (Thermo Fisher Scientific, Massachusetts, USA) according to the manufacturer's instructions.

### Colony Formation Assay

For the colony formation assay, the AGS cells were trypsinized after siRNA transfection of RNASET2 for 48 h, and then 1 × 10^3^ cells were plated in 10 cm dishes and incubated at 37°C for 12 days. Colonies were dyed with crystal violet staining solution. Cell colonies were then counted and analyzed using OpenCFU software.

### GEO and TCGA Datasets Analysis

Human GAC (cancer vs. normal) samples and clinical information, as well as their corresponding RNASET2 mRNA expression data, were obtained from Gene Expression Omnibus (GEO) datasets (http://www.ncbi.nlm.nih.gov/gds/) and The Cancer Genome Atlas (TCGA) datasets (https://cancergenome.nih.gov/). GSE7307 was applied to calculate the relative expression level of RNASET2 mRNA in different anatomic zones of human normal stomach tissues. Two independent datasets from GSE19826 (25) and GSE13911 (26) were used to calculate the relative expression level of RNASET2 mRNA in human GAC and corresponding normal gastric mucosa tissues. For GSE7307, GSE19826, and GSE13911, the intensity of probe 1556201_at was used to represent the RNASET2 mRNA expression level. For Lauren's classification analysis, 265 cases of stomach adenocarcinoma samples [TCGA, Nature 2014 (27), including 173 cases of intestinal type, 65 cases of diffuse type, 16 cases of mixed type, and 11 cases of unclassified type] and their corresponding RNASET2 mRNA expression data were extracted using cBioPortal online software (http://www.cbioportal.org). For TNM classification and survival analysis, UCSC Xena (https://xenabrowser.net/), a functional genomics explorer, was utilized to download and normalize a total of 415 cases of primary stomach adenocarcinoma (388 cases with overall survival, 366 cases with TNM classification) and their corresponding RNASET2 mRNA expression data from several TCGA datasets.

### Analysis of Genome-wide RNA Interference (RNAi) Proliferation Screening Data

The Gene Dependency Scores of 713 cancer cell lines using DEMETER2 model were obtained from the DepMap database (https://depmap.org/portal/) and filtered to include only those 547 cell lines of 26 cancer types in which RNAi-RNASET2 was detected. There were 21 gastric cancer cell lines (X2313287, AGS, ECC10, FU97, GSS, GSU, HGC27, HUG1N, IM95, KATOIII, KE39, MKN1, MKN45, MKN7, NCCSTCK140, NCIN87, NUGC3, SNU216, SNU5, SNU601, SNU719) and 34 ovarian cancer cell lines in this dataset. In DEMETER2 method, the Gene Dependency Scores were calculated from RNAi screening data and normalized using a uniform scaling and offset [applied to all cell lines (28, 29)]. The lower the score, the more likely the gene is an oncogene. In contrast, the higher the score, the more likely the gene is a tumor suppressor gene. To compare the distribution of the Gene Dependency Scores in 21 gastric cancer cell lines (test group), 34 ovarian cancer cell lines (positive control group of tumor suppressor gene), and all 547 cancer cell lines (negative control group of tumor suppressor gene), a non-parametric Wilcox rank-sum test was performed between them. A threshold of *p* < 0.05 was considered significant for all analyses.

### Analysis of Genome-wide CRISPR/Cas9 Proliferation Screening Data in GAC Cell Lines

One lakh seventy thousand six hundred and thirty-four genes knockout data for 563 cell lines of 27 primary diseases were obtained from the DepMap database (https://depmap.org/portal/) [CRISPR(Avana) Public 19Q2] and filtered to include only 19 GAC cell lines (X2313287, SH10TC, GSS, KE97, SNU719, SNU216, HGC27, NCIN87, SNU1, LMSU, MKN74, NUGC3, GSU, KE39, HS746T, FU97, SNU601, GCIY, AGS) in which RNASET2 knockout data was detected. In this database, CERES dependency score, based on data from a cell depletion assay, was used to represent the changes of cell growth after interest genes were knocked out. A score of 0 indicates a gene is not essential for cell growth; correspondingly −1 is comparable to the median of all pan-essential genes. The lower the score, the more likely the gene is an oncogene. The higher the score, the more likely the gene is a tumor suppressor gene.

### Gene Set Enrichment Analysis (GSEA)

Two hundred and sixty-five cases of stomach adenocarcinoma samples and clinical information (TCGA, Nature 2014) (27), as well as their corresponding whole genome expression profiles, were downloaded using cBioPortal software (http://www.cbioportal.org). These patients were divided into two groups according to their RNASET2 mRNA expression level [top 50%, *n* = 132: high expression group (H) vs. bottom 50%, *n* = 133: low expression group (L)]. JAVA program for GSEA (http://www.broadinstitute.org/gsea) was carried out to assess the effects of the low expression level of RNASET2 mRNA on various biological states. The curated gene sets database (c2.all.v6.1) and GO gene sets database (c5.all.v6.1) from the molecular signatures database (MsigDB) were used for enrichment analysis. One-thousand random sample permutations were carried out, and the significance threshold set at the nominal *p*-value (NOM *p*) < 0.01.

### Statistical Analysis

All statistical analyses were performed by SPSS 15.0 statistical software (SPSS, Inc., Chicago, IL, USA). The difference of RNASET2 protein positive expression rate in the various group of human tissues was analyzed by Chi-Square and Fisher's exact tests. The possible relationship between RNASET2 protein expression and the clinical pathological features was analyzed by Chi-Square and Fisher's exact tests. The correlation between RNASET2 protein and TM4SF1, S100A12, NF-κB p65, ERK, Ki67, p53 protein expression was analyzed by Spearman rank correlation analysis. Student's two-tailed *t*-test and paired *t*-test were used to compare data of RNASET2 mRNA between two groups. One-way ANOVA was used to compare data of RNASET2 mRNA among three groups or more. For survival analysis, the survival curves were calculated by the Kaplan–Meier method, and the log-rank test was used to compare them. A *p-*value of < 0.05 was set to be the significance level.

## Results

### The Diversity of RNASET2 Expression in Different Anatomic Zones of Stomach

**Figure 1:**
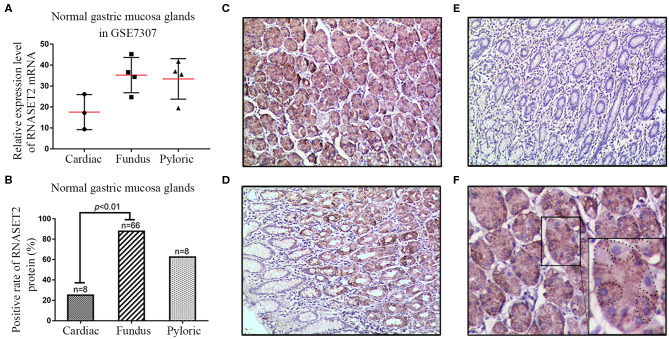
Expression and localization of RNASET2 in different anatomic zones of the stomach. **(A)** The dataset from GSE7307 showed the relative expression level of RNASET2 mRNA in fundus gland, pyloric gland, and cardiac gland of the normal gastric mucosa tissues. **(B)** Positive rate of RNASET2 protein in fundus gland, pyloric gland, and cardiac gland of normal gastric mucosa tissues (*p* < 0.01, Chi-Square and Fisher's exact tests). **(C–E)** Immunohistochemical staining of RNASET2 protein in fundus gland **(C)**, pyloric gland **(D)**, and cardiac gland **(E)** of the normal gastric mucosa; DAB staining (brown); nuclear counterstaining (hematoxylin); original magnification, ×100. **(F)** Representative tissue specimens of RNASET2 protein expression in most of the chief cells and part of the parietal cells; Dotted line areas represent the parietal cells; DAB staining (brown); nuclear counterstaining (hematoxylin); original magnification, ×400.

To investigate the expression and localization of RNASET2 in the normal gastric mucosa, an independent microarray dataset (GSE7307) containing three cases of cardiac gland tissues, four cases of fundus gland tissues, and four cases of pylorus gland tissues were firstly analyzed. The results showed that the expression of RNASET2 mRNA increased 2.002-fold in fundus glands (35.26 ± 4.209) or 1.899-fold in pylorus glands (33.44 ± 4.837) than that in cardiac glands (17.61 ± 4.826; Figure 1A). Likewise, to verify whether the RNASET2 protein is also abundant in the fundic glands, we observed the immunohistochemical staining results in the 82 cases of normal gastric mucosa tissues. The positive rate of RNASET2 protein expression was higher in fundus glands (87.88%, 58/66) and pylorus glands (62.50%, 5/8) than those of cardiac glands (25%, 2/8). The difference between cardiac glands and fundus glands was statistically significant (*p* < 0.01; Figure 1B). The positive signals of RNASET2 protein were brown in color and located in cytoplasm of the gastric mucosa epithelial cells. RNASET2 protein was mainly located in the zone of fundus, body (Figure 1C), and part of antral and pylorus mucosa (Figure 1D), while positive staining was hard to find in gastric cardiac mucosa (Figure 1E). In antral and pylorus mucosa, RNASET2 protein was mainly expressed in the inherent glands composed of fundus glands (upper right of Figure 1D), rather than in the superficial cervical mucus cells (bottom left of Figure 1D). In the fundus gland, high expression of RNASET2 protein was mainly observed in most of the chief cells and part of the parietal cells (Figure 1F).

### RNASET2 Expression Was Down-Regulated in GAC

**Figure 2:**
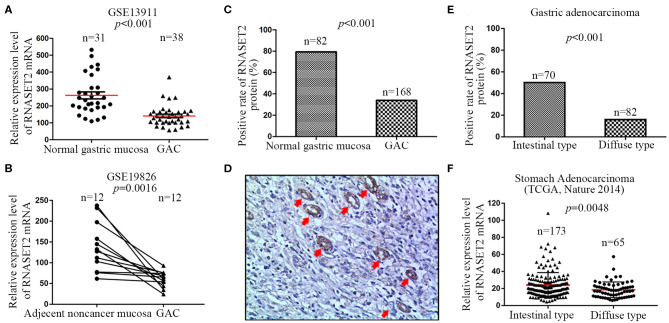
Differential expression of RNASET2 between GAC and adjacent non-cancerous or normal gastric mucosa tissues. **(A)** The dataset from GSE13911 showed the relative expression level of RNASET2 mRNA was decreased 2.29-fold in GAC tissues than that in normal gastric tissues (*p* < 0.001, Student's two-tailed *t*-test). **(B)** 12 paired GAC and adjacent non-cancerous gastric mucosa tissues from GSE19826 showed the relative expression level of RNASET2 mRNA was lower in GAC tissues than that in adjacent non-cancerous gastric tissues (*p* = 0.0016, Paired *t*-test, two-tailed). **(C)** Positive rate of RNASET2 protein in normal gastric mucosa and GAC tissues (*p* < 0.001, Chi-Square test). **(D)** Representative tissue specimens of RNASET2 protein expression in GAC and undamaged gastric mucosa glands; Red arrowhead represent undamaged gastric mucosa glands; DAB staining (brown); nuclear counterstaining (hematoxylin); original magnification, ×100. **(E)** Positive rate of RNASET2 protein in intestinal type GAC and diffuse type GAC tissues (*p* < 0.001, Chi-Square test). **(F)** TCGA stomach adenocarcinoma dataset (Nature 2014) showed the relative expression level of RNASET2 mRNA was decreased 1.30-fold in diffuse type GAC tissues than that in intestinal type GAC (*p* = 0.0048, Student's two-tailed *t*-test).

To investigate the distinction of RNASET2 between normal gastric mucosa and GAC tissues, two independent microarray datasets containing GSE13911 and GSE19826 were firstly analyzed. The results demonstrated that a significantly decreased expression of RNASET2 mRNA in GAC tissues compared with normal or adjacent non-cancerous gastric mucosa tissues (*p* < 0.001, Figures 2A,B). Thus, the expression of RNASET2 protein was further detected in 168 GAC tissues, and then compared with that in 82 normal gastric mucosa tissues collected previously. We found that RNASET2 protein positive rate was also significantly decreased in GAC (33.93%, 57/168) in comparison to normal gastric mucosa tissues (79.27%, 65/82; *p* < 0.001; Figure 2C). Representative immunohistochemical staining specimen of GAC and undamaged gastric mucosa glands was presented in Figure 2D. According to Lauren's criteria, GAC was classified into intestinal type, diffuse type, and mixed type. We found that the down-regulation of RNASET2 protein in GAC was more often present in diffuse type GAC (15.86%, 13/82) than that in intestinal type GAC (50%, 35/70; *p* < 0.001; Figure 2E). TCGA stomach adenocarcinoma data (Nature 2014, 173 cases of intestinal type and 65 cases of diffuse type) also confirmed that the expression levels of RNASET2 mRNA were lower in diffuse type GAC than that in intestinal type GAC (*p* = 0.0048; Figure 2F).

### RNASET2 Was a Biomarker for Identifying Early GAC

**Figure 3:**
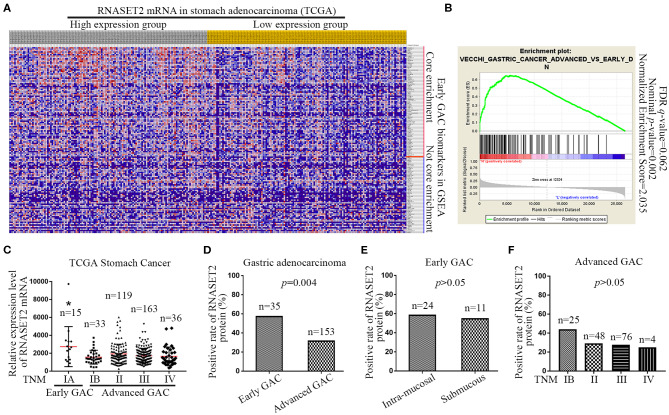
Expression of RNASET2 in early and advanced GAC tissues. **(A)** Heat map of GSEA showed that a large number of gene sets (79/134) involved in early GAC were enriched in high RNASET2 mRNA expression group; The largest values were displayed as the reddest and the smallest values were displayed as the bluest. **(B)** Enrichment plot of “VECCHI_GASTRIC_CANCER_ADVANCED_VS_EARLY_DN” showed that the normalized enrichment score was 2.035. **(C)** TCGA datasets showed the relative expression level of RNASET2 mRNA in TNM IA, IB, II, III, and IV stage (*p = 0.0014, TNM IA vs. TNM IB, II, III, or IV, ANOVA). **(D)** Positive rate of RNASET2 protein in early GAC and advanced GAC tissues (*p* = 0.004, Chi-Square test). **(E)** Positive rate of RNASET2 protein in intra-mucosal early GAC and submucous early GAC (*p* > 0.05, Chi-Square test). **(F)** Positive rate of RNASET2 protein in TNM IB, II, III, and IV stage advanced GAC (*p* > 0.05, Chi-Square test).

Next, we downloaded the entire TCGA Stomach Adenocarcinoma (TCGA, Nature 2014) dataset, including the mRNA expression value of 29,505 genes, and performed a GSEA to analyze the potential relationship between RNASET2 mRNA expression and curated gene sets (C2) in the MSigDB. The results indicated that most of (79/134) gene sets involved in early GAC (VECCHI_GASTRIC_CANCER_ADVANCED_VS_EARLY _DN) pathway were enriched in RNASET2 mRNA high expression group (Figure 3A). Enrichment score curve was presented in Figure 3B (Normalized enrichment score = 2.035, Nominal *p* = 0.002). Furthermore, 366 cases of GAC from TCGA Stomach Cancer database proved that the RNASET2 mRNA expression level was lower in advanced GAC (TNM IB, II, III, IV) than that in early GAC (TNM IA) (*p* = 0.001; Figure 3C). Thus, we further detected the expression difference of RNASET2 protein between early GAC and advanced GAC in 168 surgical resection specimens (15 TNM IA cases and 153 advanced cases) and 20 ESD specimens (TNM IA). Representative immunohistochemical staining specimens of the early GAC and advanced GAC was presented in Supplemental Figure 1. The statistical results demonstrated that the positive rate of RNASET2 protein expression was lower in advanced GAC (31.37%, 48/153) than that in early GAC (57.14%, 20/35; *p* = 0.004; Figure 3D). However, in the early GAC, no significant difference was observed between intra-mucosal GAC and submucous GAC (*p* > 0.05; Figure 3E). In the advanced GAC, there was also no significant difference among TNM IB, II, III, IV stage GAC (*p* > 0.05; Figure 3F).

### Association of RNASET2 Expression With the GAC Clinicopathologic Features and Patients' Survival

**Figure 4:**
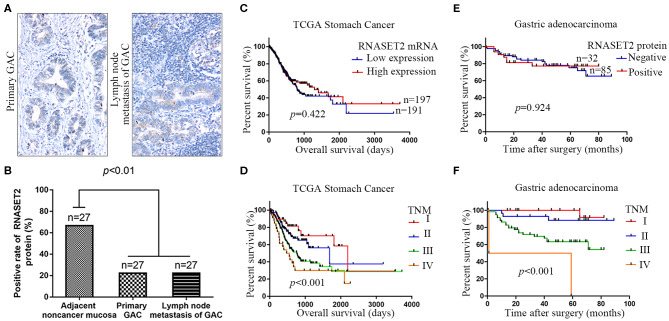
The relationship between the expression of RNASET2 and lymph nodal metastasis or patient's prognosis. **(A)** Representative tissue specimens of RNASET2 protein expression in primary GAC (left) and lymph nodal metastasis of GAC (right); DAB staining (brown); nuclear counterstaining (hematoxylin); original magnification, ×100. **(B)** Positive rate of RNASET2 protein in the primary GAC, lymph nodal metastasis of GAC, and adjacent non-cancerous gastric mucosa tissues (*p* < 0.01, Chi-Square test). **(C)** TCGA datasets showed the relationship of RNASET2 mRNA expression to overall survival (*p* = 0.422): Low expression group, *n* = 191; High expression group, *n* = 197. **(D)** TCGA datasets showed the relationship of TNM stage to overall survival (*p* < 0.001): TNM I, *n* = 49; TNM II, *n* = 119; TNM III, *n* = 163; TNM IV, *n* = 36; Log-rank test. **(E)** Relationship of RNASET2 protein expression to overall survival (*p* = 0.924): IHC score = 0 or 1 were considered to RNASET2 protein negative expression, *n* = 85, IHC score = 2 or 3 were considered to RNASET2 protein positive expression, *n* = 32. **(F)** Relationship of TNM stage to overall survival (*p* < 0.001): TNM I, *n* = 28; TNM II, *n* = 29; TNM III, *n* = 58; TNM IV, *n* = 2.

As illustrated in the Table 1, the clinicopathologic feature analysis results demonstrated that the expression of RNASET2 protein was significantly positively correlated with tumor cell differentiation (*p* < 0.001). However, there was no significant correlation of RNASET2 protein expression with the other clinicopathologic characters, such as patient age, gender, size of the tumor, depth of invasion, and lymph nodal metastasis (*p* > 0.05). In addition, we randomly selected 27 cases of advanced GAC patients with lymph nodal metastasis from another cohort, and then detected RNASET2 protein expression between the primary tumor and lymph nodal metastatic tumor in GAC. The result showed that the positive rate of RNASET2 protein expression was the same in these two groups. The positive rate of RNASET2 protein expression in the primary GAC (22.22%, 6/27) or lymph nodal metastasis of GAC (22.22%, 6/27) was significantly lower than that in the adjacent non-cancerous gastric mucosa tissues (66.67%, 18/27; *p* < 0.01; Figures 4A,B).

**Table 1:** Correlation between RNASET2 protein expression and clinicopathologic features of GAC.

**Clinicopathological features**	**All cases**	**RNASET2 protein expression**		
		**Negative**	**Positive (%)**	***p*-value^a^**
Age at diagnosis (years)				0.559
≤ 56	76	52	24 (31.58%)	
>56	92	59	33 (35.87%)	
Gender				0.491
Male	109	70	39 (35.78%)	
Female	59	41	18 (30.51%)	
Size (diameter), cm^b^				0.182
<4 cm	105	70	35 (33.33%)	
≥4 cm	52	38	14 (26.92%)	
Depth of invasion				0.312
T1a	11	5	6 (54.54%)	
T1b	11	6	5 (45.45%)	
T2	33	21	12 (36.36%)	
T3/T4	113	79	34 (30.09%)	
Nodal metastasis				0.109
N0	57	33	24 (42.11%)	
N1/N2/N3	111	78	33 (29.73%)	
Differentiation				<0.001
Well/Moderately	65	31	34 (52.31%)	
Poorly/Undifferentiated	103	80	23 (22.33%)	

a *Chi-square test*.

b *11 cases were not included due to the discrepancy of tumor size*.

To further explore the correlation between RNASET2 and the prognosis of GAC patients, we utilized TCGA Stomach Cancer database to perform survival analysis. The results revealed that RNASET2 mRNA expression was not correlated with GAC patients' survival (*p* = 0.422; Figure 4C). As a control, the correlation between TNM stage and GAC patients' survival was also analyzed in TCGA GAC samples (*p* < 0.001; Figure 4D). Next, the overall survival information for 117 GAC patients in our samples was obtained by a post-operative follow-up (Figures 4E,F). To determine the representativeness of this GAC cohort, the well-established prognostic factors such as TNM stage for the survival of the patients were firstly tested. Kaplan–Meier analysis and log-rank test showed that the decreased patients' survival was significantly correlated with later TNM stage (*p* < 0.001; Figure 4F). And then, an assessment of patients' survival showed no significant difference between RNASET2 protein positive group and negative group (*p* = 0.924; Figure 4E). Furthermore, the correlation between RNASET2 protein expression and the prognosis of advanced GAC patients or different types (diffuse type or intestinal type) of GAC patients was also evaluated. However, no statistical significance was observed (Supplemental Figure 2).

### RNASET2 Was a Cancer Passenger Gene in the GAC

**Figure 5:**
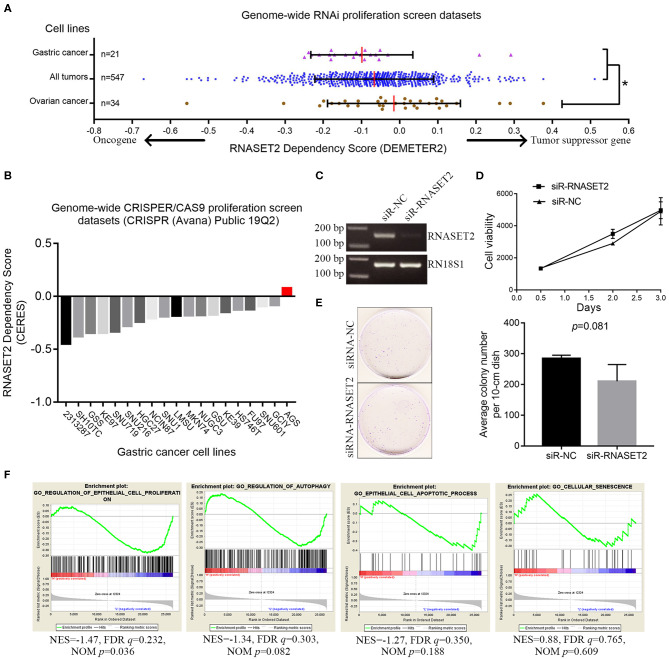
The relationship between RNASET2 and the cell growth of GAC. **(A)** The distribution of RNASET2 dependency scores (DEMETER2) in gastric cancer cell lines, ovarian cancer cell lines, and all cancer cell lines group. Red line was the median of the RNASET2 Dependency Scores in the group. *the gene dependency scores in ovarian cancer were significantly higher than that in gastric cancer and all cancer cell line groups (*p* < 0.05). **(B)** The CERES dependency score of RNASET2 in 19 GAC cell lines. Red bar was the CERES dependency score of RNASET2 in AGS. A score of 0 indicates a gene is not essential for cell growth; correspondingly −1 is comparable to the median of all pan-essential genes. **(C)** RNASET2 mRNA expression were detected by the reverse transcription PCR after transient (48 h) siRNA transfection of RNASET2 or negative control (NC). **(D)** Cell viability was detected by AlamarBlue assay after transient (12, 48, and 72 h) siRNA transfection of RNASET2 or NC, *p* > 0.05, *n* = 3. **(E)** Clonogenicity of AGS cells was detected by colony formation assay after siRNA transfection of RNASET2 or NC for 12 days, *p* > 0.05, *n* = 3. **(F)** GSEA enrichment plots showed that the expression change of RNASET2 mRNA was not enriched in gene sets of “epithelial cell proliferation,” “regulation of autophagy,” “epithelial cell apoptotic process,” and “cellular senescence” (NOM *p* > 0.01).

Our above clinical sample studies only demonstrated a correlation between RNASET2 and GAC. However, it is still unclear whether the change of RNASET2 expression is the cause (driver gene: oncogene or tumor suppressor gene) or the consequence (passenger gene) of GAC occurrence. Therefore, gene knockdown data from a previously published genome-wide RNAi screening database for 547 cancer cell lines were analyzed (30). According to DEMETER2 method in this database, the gene dependency scores were calculated from the RNAi screening data. The lower the gene dependency score, the greater probability that the gene is an oncogene. In contrast, the higher the gene dependency score, the greater probability that the gene is a tumor suppressor gene. RNASET2 was reported to be a tumor suppressor gene in ovarian cancer (12). Therefore, ovarian cancer was set as positive control for tumor suppressor gene. Then, we compared the differences of gene dependency scores of the three groups such as ovarian cancer cell lines group (*n* = 34), all cancer cell lines group (*n* = 547), and GAC cell lines group (*n* = 21). The Wilcox rank-sum test results showed that the gene dependency scores in GAC group were significantly lower than that in ovarian cancer group and all cancer cell lines group (Figure 5A). It means that RNASET2 might not be served as a tumor suppressor gene in GAC. Meanwhile, the gene dependency scores of GAC (median: −0.113) were >-0.5, excluding RNASET2 is an oncogene.

Secondly, genes knockout data for 563 cell lines from another published genome-wide CRISPR/Cas9 proliferation screening database (https://depmap.org/portal) were analyzed. In this database, a lower CERES dependency score indicates a higher likelihood that the gene is essential for cell growth in a given cell line. Similar to DEMETER2 method, the lower the CERES dependency score, the greater probability that the gene is an oncogene. The CERES dependency score of RNASET2 gene data in 19 GAC cell lines were extracted. The results showed that the CERES dependency scores of 19 GAC cell lines were all >-1. The lowest score was −0.449, which came from 2,313,287 GAC cell line (Figure 5B). This result indicated RNASET2 was not an oncogene (essential gene for cell growth) in 19 GAC cell lines. Finally, to further determine whether RNASET2 is a tumor suppressor gene or a passenger gene, we selected the AGS cell line, which had a highest score (CERES = 0.077) in 19 GAC cell lines, to perform a serials of validation experiments. Reverse transcription PCR result confirmed that RNASET2 expression in AGS cell was significantly inhibited by chemically synthetic small interfering RNA (siRNA) (Figure 5C). AlamarBlue cell viability assay and colony formation assay demonstrated that the growth of AGS cells were not significantly enhanced after the expression of RNASET2 decreased (*p* > 0.05; Figures 5D,E). GSEA results also showed that RNASET2 of GAC was not involved in tumor cell viability related biological processes, such as “epithelial cell proliferation,” “regulation of autophagy,” “epithelial cell apoptotic process,” and “cellular senescence” (NOM *p* > 0.01; Figure 5F). Therefore, we speculated that RNASET2 is a passenger gene in GAC.

### RNASET2 didn't Exert an Anti-angiogenic Effect in GAC

**Figure 6:**
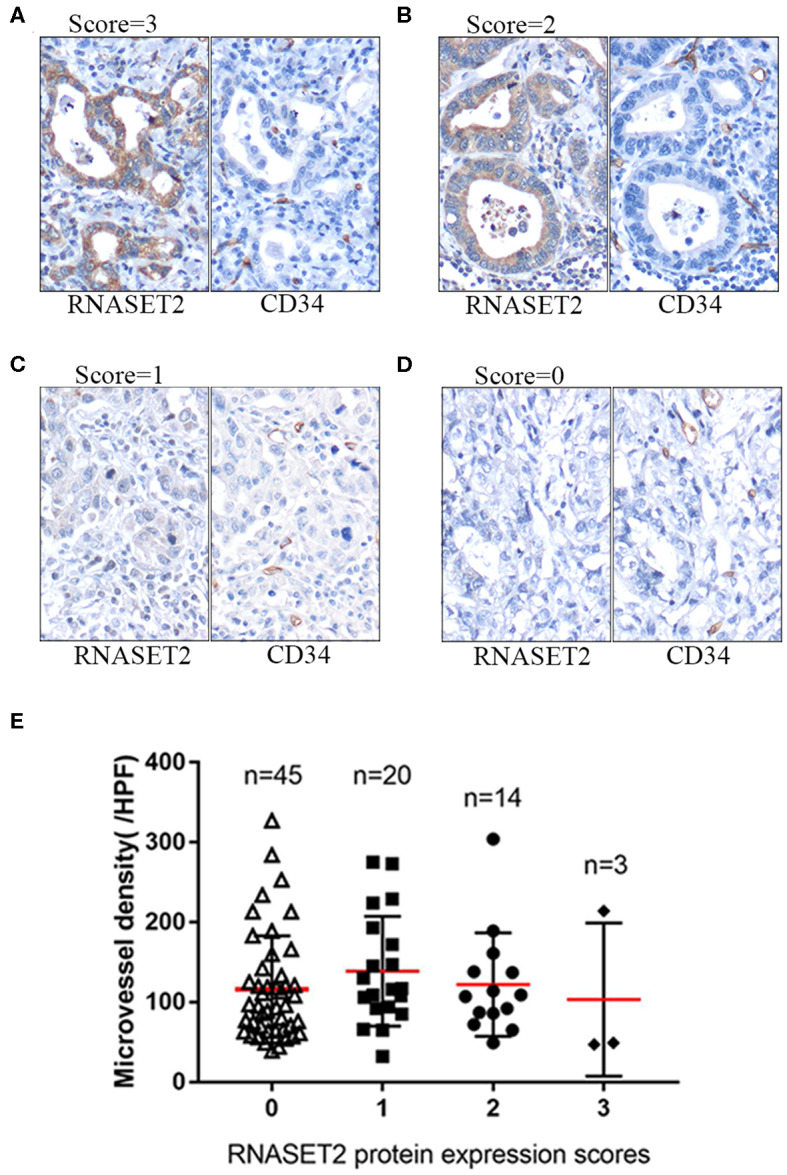
The expression of RNASET2 and CD34 proteins in GAC tissues. **(A–D)** Immunohistochemical staining of RNASET2 and CD34 proteins in representative tissue specimens. **(A)** RNASET2 Score = 3, **(B)** Score = 2, **(C)** Score = 1, and **(D)** Score = 0. The microvessels were labeled by the antibody of CD34. DAB staining (brown); nuclear counterstaining (hematoxylin); original magnification, ×400. **(E)** The relative density of microvessels were counted in the different scores of RNASET2 protein expression (*p* = 0.619, ANOVA).

We used the antibody of CD34 to label the microvessel in 82 cases of GAC and analyzed the relationship between the expression of RNASET2 protein and angiogenesis of GAC. The results indicated that there was no significant correlation between RNASET2 protein expression and microvessel density (*p* > 0.05). The microvessel density of RNASET2 protein score of 0, 1, 2, and 3 were 116.24 ± 66.92, 138.9 ± 68.57, 122.14 ± 64.79, and 103.33 ± 95.85, respectively (Figure 6).

### Correlation Between RNASET2 Protein and GAC-associated Markers

Finally, the relationships between GAC-related proteins reported in our previous studies and RNASET2 in current study were further analyzed. As illustrated in Table 2, positive correlation was observed between RNASET2 and TM4SF1 protein (*p* < 0.001). In addition, the relationship between RNASET2 protein and other GAC-associated markers, such as S100A12, ERK, NF-κB p65, p53, and Ki67 were further explored. However, there was no correlation between RNASET2 protein expression and these proteins (*p* > 0.05; Table 2).

**Table 2:** Correlation between the expression of RNASET2 and other GAC-related proteins.

**Proteins**	**All cases^b^**	**RNASET2 protein expression**			
		**Negative**	**Positive (%)**	***r-*value^a^**	***p*-value^a^**
TM4SF1				0.472	0.000
Positive	12	4	8 (66.67%)		
Negative	72	63	9 (12.50%)		
S100A12				0.194	0.083
Positive	61	29	32 (52.46%)		
Negative	20	14	6 (30.00%)		
NF-κB p65				−0.069	0.548
Positive	13	8	5 (38.46%)		
Negative	65	34	31 (47.69%)		
ERK				0.029	0.799
Positive	60	31	29 (48.33%)		
Negative	20	11	9 (45.00%)		
Ki67				0.103	0.364
Positive	65	33	32 (49.23%)		
Negative	14	9	5 (35.71%)		
P53				0.038	0.743
Positive	35	17	18 (51.43%)		
Negative	42	22	20 (47.62%)		

a *Spearman rank correlation test*.

b *Protein expression levels were detected in 84 GAC tissues (some cases were absent)*.

## Discussion

Stomach cancer is the fourth most common cancer worldwide. Over 90% of the cancers that occur in the stomach are GAC (31). In this study, RNASET2 mRNA and protein expression were down-regulated in GAC tissues, suggesting that lower expression of RNASET2 was associated with gastric carcinogenesis. In order to exclude the possible bias caused by sample selection, two groups of GAC patients from different cohorts were enrolled in this study. The first group was unpaired samples which included GAC tissues and normal gastric mucosa tissues. The second group was paired samples which included GAC tissues and adjacent non-cancerous gastric mucosa tissues. The results showed that the expression of RNASET2 in both normal gastric mucosa and adjacent non-cancerous gastric mucosa tissues were higher than that in GAC tissues. Besides, the down-regulation of RNASET2 mRNA and protein was more common in diffuse type than that in intestinal type GAC, which indicated that the low expression of RNASET2 was more closely related with the tumorigenesis of diffuse type GAC.

GAC is highly curable when diagnosed and treated in the early stage (32). Therefore, it is vital to identify novel biomarkers in early GAC. In the present study, the early GAC (TNM stage IA) is histologically defined as GAC confined to the lamina propria, muscularies mucosae, or submucosa, without the presence of lymph node metastasis and distant metastasis (33). Others GAC (TNM IB, II, III, IV) are placed into advanced GAC. We observed that the expression of RNASET2 mRNA and protein were higher in early GAC than that in advanced GAC. GSEA analysis showed that RNASET2 was involved in the signal pathway of early GAC. These results confirmed that RNASET2 could be regarded as a good biomarker to identify the early stage of GAC. On the contrary, no significant correlation was detected between RNASET2 protein expression and several clinicopathologic characters of advanced GAC, such as depth of invasion, lymph nodal metastasis, and TNM stage (TNM IB, II, III, IV). Furthermore, we also found that the positive rate of RNASET2 protein expression was the same between the primary tumors and lymph nodal metastatic tumors in GAC. These results indicated that RNASET2 might not be a good biomarker to estimate the prognosis of GAC patients. Kaplan–Meier analysis confirmed this inference that there was no significant correlation between RNASET2 expression and patient's prognosis.

In addition to its ability to degrade RNA, RNASET2 has been reported to have anti-angiogenic and anti-tumorigenic effects in some tumors. In order to elucidate the role of RNASET2 in GAC, we performed the cell viability and microvessel density studies, as well as bioinformatics analysis. Genome-wide RNAi proliferation screening and genome-wide CRISPR/Cas9 proliferation screening are beneficial to identify which genes are essential for cell survival and growth. The difference between the two methods is that the expression of RNASET2 in the former is knocked down by shRNA at the level of messenger RNA, while the expression of RNASET2 in the latter is knocked out by CRISPR/Cas9 at the level of DNA. The DepMap database and DEMETER2 model provided a simple and effective method for defining and predicting genes that are essential for cell viability (29). In genome-wide RNAi proliferation screening dataset, we set RNASET2 dependency score of ovarian cancer cell lines as a positive control due to RNASET2 was a known tumor suppressor gene in ovarian cancer. In genome-wide CRISPR/Cas9 proliferation screening dataset, we chose AGS cell line for further validation because AGS had the highest CERES dependence score. If RNASET2 is not a tumor suppressor gene in AGS, then RNASET2 is also not a tumor suppressor gene in other 18 GAC cell lines. Further cell viability assay and colony formation assay demonstrated that RNASET2 was indeed not a tumor suppressor gene in AGS. All these results indicated that the reduction in RNASET2 expression was the consequence of GAC instead of the cause. GSEA analysis showed that RNASET2 expression was not associated with tumor cell viability related biological processes, also suggesting that RNASET2 was a cancer passenger gene in GAC, rather than a driver gene. Next, to verify whether RNASET2 could play an anti-angiogenic role in GAC, we used the antibody of CD34 to label the microvessel in the GAC tissues. Unfortunately, there was no significant correlation between RNASET2 protein expression and microvessel density, suggesting that RNASET2 also could not exert angiogenesis effects in the GAC.

Human normal gastric mucosa consists of cardiac glands, fundus glands, and pyloric glands. The cardiac glands secreting mucus are found in the cardia of the stomach. The fundus glands secreting hydrochloric acid and pepsinogen are located in the fundus and body of the stomach. The pyloric glands secreting gastrin and mucus are present in the antrum and pylorus of the stomach (34). Among them, the fundus glands and part of the pylorus glands (inherent glands) constitute the main functional unit of the stomach. In the present study, high expression of RNASET2 protein was mainly found in the zone of fundus, body, and part of antral and pylorus mucosa, indicating that the normal gastric mucosa can produce a large number of RNASET2. RNASET2 is a member of a family of transfer-type endonucleases that cleave RNA substrates without strict base preference at acidic pH value (5). Thus, a gastric acidic environment is suitable for RNASET2 to exert its endonucleases function to cleave RNA substrates. Combining cell viability assay, colony formation assay, microvessel density, and bioinformatics analysis, it is reasonable to speculate that RNASET2 only plays the role of its ribonuclease capacity in GAC, rather than anti-angiogenic and anti-tumorigenic effects. The change of RNASET2 expression is a consequence of GAC, rather than a cause.

Transmembrane-4-L-six-family member-1 (TM4SF1) is a small plasma membrane glycoprotein that regulates cell proliferation and motility (35). S100A12 belongs to the S100 family of calcium-binding protein (36). Our previous studies reported that both TM4SF1 and S100A12 proteins were tumor suppressor genes in GAC (23, 24). In this study, we found a positive correlation between the expression of RNASET2 and TM4SF1 protein. However, no significant correlations between RNASET2 and S100A12 protein was observed. Future studies will determine whether the down-regulation of RNASET2 affects the expression of TM4SF1. In addition, it is well-documented that p53 is a crucial tumor suppressor in GAC (37). NF-κB p65 is a kind of transcription factor participating in the occurrence and progression of GAC (38). ERK belongs to the MAPK signaling pathways, which is essential for GAC cell proliferation and migration (39). Ki67 protein is present in the G1 phase, S phase, G2 phase, and M phase of the cell cycle, which is closely related to cell proliferation (40). Accordingly, the correlations between RNASET2 protein with p53, NF-κB p65, ERK, and Ki67 were also explored. However, no significant correlations between RNASET2 protein and these proteins were found.

## Conclusion

In summary, we first found that RNASET2 was abundant in the fundus glands and pylorus glands of the normal gastric mucosa. Then, the down-regulation of RNASET2 mRNA and protein were observed in GAC tissues. However, we considered that the decrease of RNASET2 expression was only the consequence of GAC, instead of the driver. The expression of RNASET2 in GAC was not related to angiogenesis, lymph nodal metastasis, and patients' prognosis. Furthermore, most of gene sets involved in the early GAC pathway were enriched in the RNASET2 mRNA high expression group. The expression of RNASET2 in early GAC was higher than that in advanced GAC. Thus, we thought that RNASET2 could be regarded as a good biomarker for identifying the early GAC.

## Data Availability Statement

Publicly available datasets were analyzed in this study. This data can be found here: The Cancer Genome Atlas (https://portal.gdc.cancer.gov/), NCBI Gene Expression Omnibus (GSE7307, GSE19826, GSE13911).

## Ethics Statement

The study was approved by the ethics committee of Renmin Hospital of the Wuhan University and was performed in accordance with the ethical standards laid down in the 1964 Declaration of Helsinki and its later amendments.

## Author Contributions

ZZ and XX conceived and designed the study. ZZ, XZ, and DL performed the research. ZZ and LG wrote the paper. ZZ and JY evaluated the immunohistochemical staining results. JL and LG analyzed the data. All authors contributed to manuscript revision, read, and approved the submitted version.

## Conflict of Interest

The authors declare that the research was conducted in the absence of any commercial or financial relationships that could be construed as a potential conflict of interest.

## Supplementary Material

Supplemental Figure 1 Representative tissue specimens of RNASET2 protein expression in early GAC and advanced GAC. **(A)** Positive expression of RNASET2 protein in the early GAC. The tumor is confined to the lamina propria mucosa. **(B)** Negative expression of RNASET2 protein in the advanced GAC. Tumor cells invade the muscularis of gastric wall. DAB staining (brown); nuclear counterstaining (hematoxylin); original magnification, ×40.

Supplemental Figure 2 The relationship between the expression of RNASET2 and the prognosis of advanced GAC patients or different types of GAC patients. **(A)** TCGA datasets showed the relationship of RNASET2 mRNA expression to overall survival of advanced gastric adenocarcinoma (*p* = 0.535): Low expression group, *n* = 185; High expression group, *n* = 188. **(B)** TCGA datasets showed the relationship of RNASET2 mRNA expression to overall survival of intestinal-type gastric adenocarcinoma (*p* = 0.174): Low expression group, *n* = 79; High expression group, *n* = 89. **(C)** TCGA datasets showed the relationship of RNASET2 mRNA expression to overall survival of diffuse-type gastric adenocarcinoma (*p* = 0.839): Low expression group, *n* = 41; High expression group, *n* = 34. **(D)** Relationship of RNASET2 protein expression to overall survival of advanced gastric adenocarcinoma (*p* = 0.711): RNASET2 protein negative group, *n* = 80, RNASET2 protein positive group, *n* = 27. **(E)** Relationship of RNASET2 protein expression to overall survival of intestinal-type gastric adenocarcinoma (*p* = 0.172): RNASET2 protein negative group, *n* = 25, RNASET2 protein positive group, *n* = 21. **(F)** Relationship of RNASET2 protein expression to overall survival of diffuse-type gastric adenocarcinoma (*p* = 0.552): RNASET2 protein negative group, *n* = 55, RNASET2 protein positive group, *n* = 6.
