# Supplementary figures and images for: Expression, Location, Clinical Implication, and Bioinformatics Analysis of RNASET2 in Gastric Adenocarcinoma
Source: Front Oncol. 2020 May 22;10:836. doi: 10.3389/fonc.2020.00836 (PMC7256199; doi:10.3389/fonc.2020.00836)

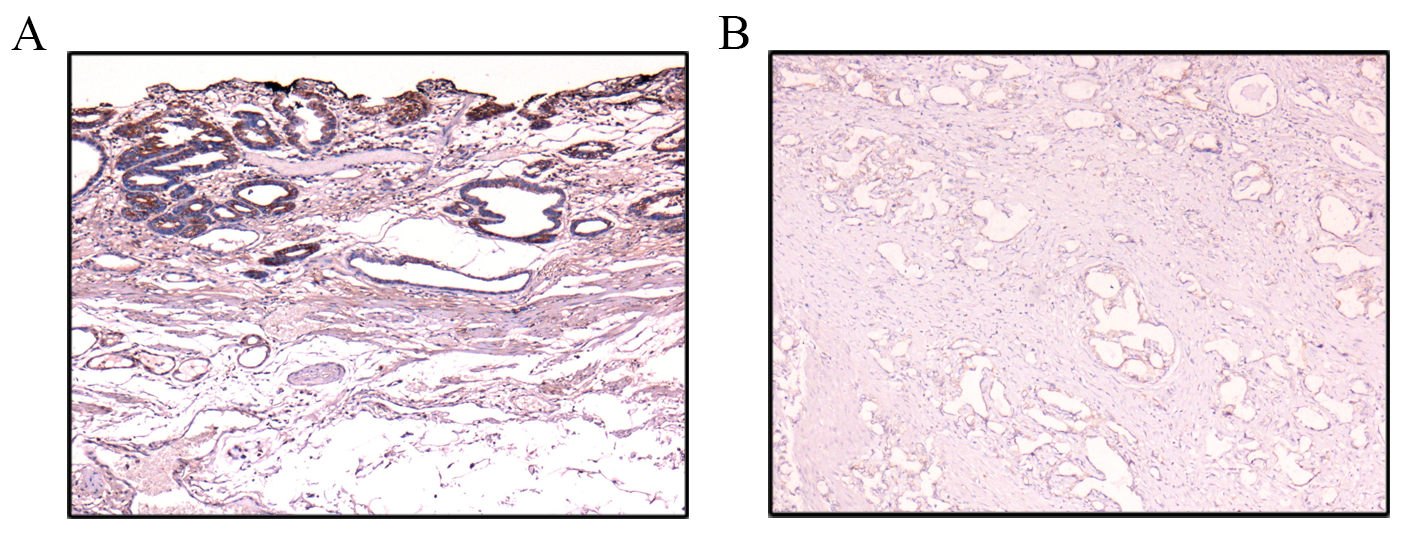

Supplement: Supplemental Figure 1 — Representative tissue specimens of RNASET2 protein expression in early GAC and advanced GAC. (A) Positive expression of RNASET2 protein in the early GAC. The tumor is confined to the lamina propria mucosa. (B) Negative expression of RNASET2 protein in the advanced GAC. Tumor cells invade the muscularis of gastric wall. DAB staining (brown); nuclear counterstaining (hematoxylin); original magnification, ×40. [file Image_1.TIF]

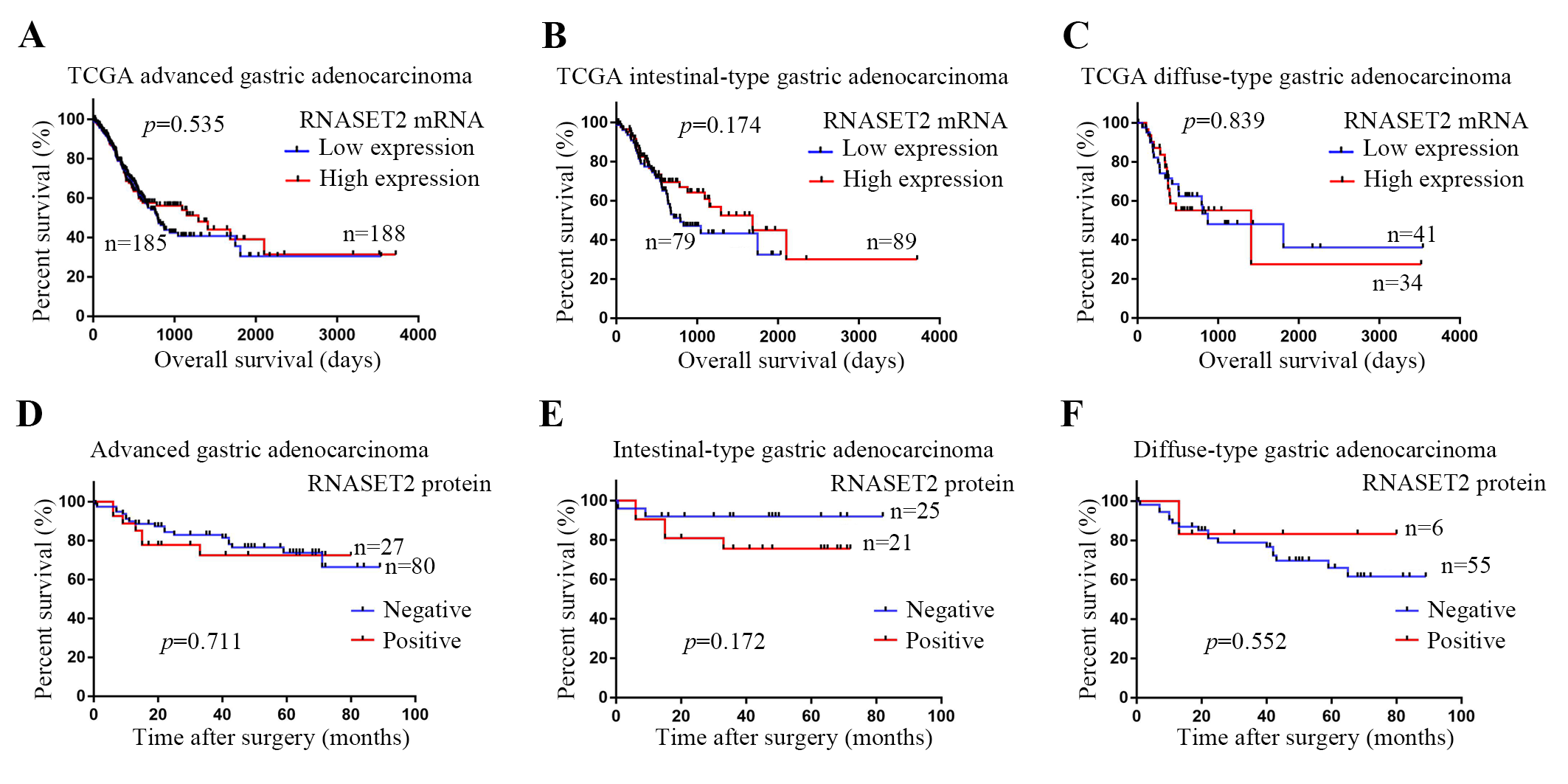

Supplement: Supplemental Figure 2 — The relationship between the expression of RNASET2 and the prognosis of advanced GAC patients or different types of GAC patients. (A) TCGA datasets showed the relationship of RNASET2 mRNA expression to overall survival of advanced gastric adenocarcinoma (p = 0.535): Low expression group, n = 185; High expression group, n = 188. (B) TCGA datasets showed the relationship of RNASET2 mRNA expression to overall survival of intestinal-type gastric adenocarcinoma (p = 0.174): Low expression group, n = 79; High expression group, n = 89. (C) TCGA datasets showed the relationship of RNASET2 mRNA expression to overall survival of diffuse-type gastric adenocarcinoma (p = 0.839): Low expression group, n = 41; High expression group, n = 34. (D) Relationship of RNASET2 protein expression to overall survival of advanced gastric adenocarcinoma (p = 0.711): RNASET2 protein negative group, n = 80, RNASET2 protein positive group, n = 27. (E) Relationship of RNASET2 protein expression to overall survival of intestinal-type gastric adenocarcinoma (p = 0.172): RNASET2 protein negative group, n = 25, RNASET2 protein positive group, n = 21. (F) Relationship of RNASET2 protein expression to overall survival of diffuse-type gastric adenocarcinoma (p = 0.552): RNASET2 protein negative group, n = 55, RNASET2 protein positive group, n = 6. [file Image_2.TIF]
